# Data report on three datasets: Mortality patterns between agricultural and non-agricultural ward areas

**DOI:** 10.3389/fgene.2022.953167

**Published:** 2023-01-04

**Authors:** Kelly Trearty, Brendan Bunting, John Mallett

**Affiliations:** Department of Psychology, Ulster University, Coleraine, United Kingdom

**Keywords:** death trends, aggregated data, area-level data, farm census, population census, Northern Ireland, Northern Ireland Neighbourhood Service (NINIS), agricultural versus non-agricultural wards

## Abstract

The health of the farming community in Northern Ireland (NI) requires further research as previous mortality studies have reported contradictory results regarding farmers’ health outcomes compared against other occupations and the general population. This study collated the NINIS area-level farm census with the population census information across 582 non-overlapping wards of NI to compile three mortality datasets (2001, 2011, and pooled dataset) (NISRA 2019). These datasets allow future researchers to investigate the influence of demographic, farming, and economic predictors on all-cause mortality at the ward level. The 2001 and 2011 mortality datasets were compiled for cross-sectional analyses and subsequently pooled for longitudinal analyses. Findings from these datasets will provide evidence of the influence of Farming Intensity scores influence on death risk within the wards for future researchers to utilise. This data report will aid in the understanding of socio-ecological variables’ additive contribution to the risk of death at the ward level within NI. This data report is of interest to the One Health research community as it standardises the environment−human−animal data to pave the way towards a new One Health research paradigm. For example, future researchers can use this nationally representative data to investigate whether agriculturally saturated wards have a higher mortality risk than non-agriculturally based wards of NI.

## Introduction

Agricultural workers have the highest work intensification of all occupations as they merge home-life with work-life and in principle are at their enterprise 365 days per year (Furey et al., 2016). The health of the farming community requires further research as previous mortality studies have reported contradictory results regarding farmers’ health outcomes compared against other occupations and the general population (Stiernström et al., 2001; Smyth et al., 2012; Cushen et al., 2016). It is still unclear which underlying mechanisms are increasing farmers’ mortality rates; therefore, this study’s compilation of the three datasets allows for various types of analyses and aims to fill some of this research gap.

The economy of Northern Ireland (NI) is dominated by the agricultural industry, as farming sectors employ approximately 1.2 million hectares or three-quarters of its acreage (NISRA 2019). In addition, NI’s physical landscape is diverse, ranging from high mountains and fertile plains to a jagged coastline, making it ideal for accommodating a vast range of agricultural activities (Abson et al., 2013). The number of NI farmers (farmers, spouses, and other farm workers) in 2001 (n = 56,362) decreased by 17% in 2011 (n = 46,848) (NISRA 2019), thus following global trends in farm number reductions (Smyth et al., 2012).

Furthermore, farmers aged between 16 and 64 years old accounted for 10% in 2001 and 7% in 2011 of the economically active NI population (HSENI, 2019b; NISRA 2019). The number of deaths in NI decreased by 2% between 2001 (n = 14,513) and 2011 (n = 14,204) (NISRA 2019). Ireland’s fatality rate in agriculture, forestry, and fishing is 5–7 times greater than any other Irish economic sector (Phelan 2018; Health and Safety Authority 2021). Farming had an average of eight on-farm deaths per year in NI until 2010; however, this increased by 50% to 12 fatalities in 2011–2012 and continues to increase (HSENI, 2019a).

Building on these statistics and the recommendations of previous research (Havelka et al., 2009; Som 2010; Allik et al., 2016; Wilkinson et al., 2016), this study linked the 2001 and 2011 population census with the farm census information across 582 non-overlapping wards of NI (NISRA 2013). This was approached by utilising the Northern Ireland Neighbourhood Information Service (NINIS) online repository to compile three mortality datasets (2001, 2011, and pooled datasets) (NISRA 2019). The current study’s database set-up emulated that of Luke and Krauss (2004), converting area-level variables into the proportion of their summed total for ease of interpretation. Findings from these datasets will provide evidence of the influence of Farming Intensity scores on death risk within wards for future researchers to utilise, and will aid in the understanding of socio-ecological variables and their additive contribution to the risk of deaths at the ward level within NI.

## Data and methods

The NINIS is a web-based service that hosts nationally representative population census and farm census information within its open-data portal (NISRA, 2019). These data were publicly available, and in January of 2019, ethical approval was granted by Ulster University Research Ethics Committee.

Table 1 identifies the origin index codes for the underlying data of 2001 and 2011 datasets (NISRA 2019). This study downloaded each variable 1) at the ward level, 2) in count form, and 3) for the census years of 2001 and 2011. Ten of the 17 variables were transformed to emulate a dichotomized measure (*age, males, below degree, living alone, limiting long-term illness, unpaid care, full-time workers, farmers,* and *grass*). Building on the previous research (Ruiz-Martinez et al., 2015), Farming Intensity was computed as a composite factor score to assess agricultural activity within each ward, using six indicator items from the farm census (farms, farmers, grass, cattle, pigs, and poultry) (NISRA 2019).

**TABLE 1:** Themes, subsets, names, and codes of raw variables extracted from the NINIS website to create 2001 and 2011 mortality datasets.

Theme	Subset	Variable	Codes	
			2011	2001
Population	Deaths	Deaths	2011	2001
Census	Demography	Ward population	QS103NI	KS02
		Age	QS103NI	KS02
		Gender	QS105NI	KS01
		Marital status	KS103NI	KS04
	Qualifications	Qualifications and students	KS501NI	KS13
	Labour market	Hours worked	DT602NI	UV041
	Health	Unpaid care	QS301NI	UV021
		Limiting long-term illness	QS303NI	UV022
Agriculture	Farm census	Farms	2011	2001
		Area farmed	2011	2001
		Grass	2011	2001
		Cattle	2011	2001
		Pigs	2011	2001
		Poultry	2011	2001
		Agricultural labour	2011	2001
		Average standard output/standard gross margin	2010	2001
People and places	Community	Ward size (hectares)	2008	2008
Deprivation	NIMDM	Northern Ireland Multiple Deprivation Measure	2010	2005

*Note*: *codes* provide unique reference codes for each variable per year. *Deprivation* reflected the overall weighed score of its seven deprivation domains: i) income, ii) employment, iii) health, iv) education, v) proximity to services, vi) environment, and vii) crime.

***Deaths:*** The outcome variable was *all-cause mortality,* which represented the total number of *deaths* observed over a 12-month period. The causes of death included in this mortality variable were as follows: malignant neoplasms C00-C97 (e.g. cancers, etc.); circulatory diseases I00-I97 (e.g. heart disease, stroke, blood clots, etc.); respiratory diseases J00-J99 (e.g. pneumonia, asthma, bronchitis, influenza, etc.); and external causes V01-Y98, X60- X84, Y10-Y34, Y87.0, Y87.2 (e.g. road traffic accidents, falls, suicide, undetermined intent, fires, poisoning, assault etc.). The *death* variable for each ward remained in its raw form, as a count.

***Ward Population:*** The 582 wards contained different population sizes ranging from approximately 740–9,500 residents. To operationalise its usefulness, the *natural log of the population* was calculated from the *ward population* variable (McGranahan 1999). This study expected wards with larger *natural log of the population* scores to be at a higher risk of *death* than wards with lower *natural log of the population* scores.

***Age:*** Derived from the date of birth question, an individual’s *age* was their age on their last birthday by that census day. *Age* was available from the NINIS databank in 2011 as a continuum (0–100+ year-olds in single years) and in 2001 as seven structured age group variables (categorised at different life stages). It was compiled into those aged *below 64 years* and *over 65 years.* This study hypothesised that wards with larger amounts of residents aged *65 to 100+ years* were at a higher risk of *death* than wards with higher amounts of *below 64-year-olds* (Doebler & Glasgow 2016; Lee et al., 2017; van Doorn et al., 2018). *Age* was converted into the proportion of *65 to 100+ year-olds* in a ward relative to the total resident population per ward.

***Gender*** was self-reported as *male* or *female* on the census day. This study postulated that wards with larger amounts of *male* inhabitants were at a higher risk of *mortality* than wards with higher amounts of *females*. The number of *males* per ward was converted into their proportion relative to the total resident population per ward.

***Marital Status*** represented a person’s legal marital status on the census day. It was dichotomized into *living alone* (summed: single, separated, divorced, and widowed) and *cohabitating* (summed: married, remarried, and same sex civil partnership). This study posited that wards with larger amounts of people *living alone* were at a higher risk of *death* than wards with higher amounts of dwellers *cohabitating*. The number of those *living alone* was converted into its proportion relative to the residents 16 years and older per ward.

***Qualifications*** represented the highest level of professional and vocational education one obtained by the census day. It was dichotomised into *below degree* (summed: no qualifications, level 1, level 2, apprenticeship, level 3, and others) and *above degree* (level 4 and level 5). This study theorized that wards with larger amounts of *below degree* qualifications were at a higher risk of *mortality* than wards with higher amounts of *above degree* qualifications. The total number of individuals with *below degree* qualifications was converted into its proportion relative to residents over 16 years old per ward.

***Limiting long-term illness (LLTI)***: It was self-reported on whether an individual had an *LLTI* or disability lasting or expected to last a minimum of 12 months from the census day. It was used to create *no LLTIs* and *yes LLTIs present* (summed: daily activities limited a little; daily activities limited a lot). The eleven types of *LLTIs* encapsulated within this variable were a combination of physical and mental health conditions. The six physical health issues were: long-term pain; mobility issues; deaf or partial hearing; blind or visually impaired; breathing issues; and chronic illness (e.g. cancer, diabetes, etc.). The five mental health issues were: communication difficulties; mental health issues (e.g. depression, schizophrenia, etc.); memory loss issues; learning or behavioural difficulties; and other conditions. This study presupposed that wards with larger amounts of residents with *LLTIs* were at a higher risk of *death* than wards with higher amounts of residents with *no LLTIs*. The number of people with an *LLTI* was converted into its proportion relative to the total number of residents per ward.

***Unpaid carers*** represented the number of hours one provides unpaid care to a care recipient (family members, friends, neighbours, and others) weekly because of their poor physical/mental health or old age. It was dichotomised into *0 h (no care provided)* and *1 or more hours* (summed: 1–19 h, 20–49 h, and 50+ hours) of unpaid care provided weekly*.* This study considered that wards with larger amounts of *unpaid carers* were at a higher risk of *mortality* than wards with higher amounts of *no unpaid carers*. The total number of people providing *unpaid care (1 or more hours)* was converted into its proportion relative to the total residents per ward.

***Hours worked weekly*** represented the number of individuals aged between 16 and 74 years who worked the week before the census in their main job, including paid and unpaid overtime. It was dichotomised into *part-time workers* (summed: 1–15 h; 16–30 h) and *full-time workers* (summed: 31–48 h; 49+ hours). This study regarded that wards with larger amounts of inhabitants working *full-time hours* were at a higher risk of *death* than wards with higher amounts of *part-time workers*. The total number of people working *full-time hours* was converted into its proportion relative to the employed residents aged 16–74 years per ward.

***Northern Ireland Multiple Deprivation Measure (NIMDM)*** reflected the overall weighed score of its seven deprivation domains. Each of the seven dimensions had different influences relating to an area’s disadvantage, such as 1) income (25%); 2) employment (25%); 3) health (15%); 4) education (15%); 5) proximity to services (10%); 6) environment (5%); and 7) crime (5%). NIMDM provided an indication of the spatial rank order for each area from the most deprived (1) to the least deprived (582) ward. This measure was able to identify if one area had higher/lower deprivation than another, but it was not able to quantify how much more/less deprived one area was to another area. This study surmised that wards with lower *NIMDM* rankings (more deprivation in that area) were at a higher risk of *mortality* than those with higher *NIMDM* rankings (less deprivation in that area). The overall 2005 and 2010 NIMDM deprivation rankings were utilised as they corresponded with the census years under analysis. NIMDM remained unchanged in their raw numerical form.

***Average standard gross margin (SGM)*** represented the annual farming profit earned from arable and/or livestock production per ward. The average SGM changed in 2011 to the average standard output (SO) within the farm census. Therefore, an approximate equivalence was assumed by including the 2001 and 2010 *SGM* information within datasets as a proxy for farm profit. The SGM represented the average profit earned from farm production within each ward (output minus production costs) and was expressed in European Size Unit (or euros). One SGM unit represented €1,200, which can be used to indicate the economic size of farming outputs within wards (NISRA, 2019). This study expected that wards with lower *farming profit* were at a higher risk of *death* than wards with higher *farming profit*. The 2001 and 2010 variables remained unchanged in their count form per ward.

***Farms*** represented the total number of active farm holdings per ward from the farm census. Active farms were defined as spatial units encapsulating 1+ hectare(s) of farmland, used for crops and/or livestock production. This study hypothesised that wards with larger amounts of *farms* were at a higher risk of *death* than wards with no farms. *Farms* remained as a count per ward.

***Agricultural labour (farmers)*** represented the total number of active farmers (16–100+ years old) employed within the agricultural industry for 20 or more weeks (39%) per year, from the farm census. This study merged farmers, spouses, and other workers to create the summed total of *agricultural labourers (or farmers)* per ward*.* This study postulated that wards with larger amounts of *farmers* were at a higher risk of *death* than wards with no farmers. The total number of *farmers* was converted into its proportion relative to the total population (16–100+ years old) per ward.

***Grass:*** NINIS defines the farming practice of producing *grass* within the farm census as the total grass in hectares, excluding rough grazing, farm woodlands, and non-agricultural land. This study posited that wards with larger amounts of *grass-based farming* were at a higher risk of *death* than wards with no grass farming. It was converted into the proportion of *grass* hectares relative to the *total hectare area farmed* per ward.

***Cattle*** represented the total number of cattle in each ward, from the Farm Census. This study theorised that wards with larger amounts of *cattle farming* were at a higher risk of *death* than wards with no *cattle*.

***Pigs*** represented the total number of pigs per ward, from the farm census. This study presupposed that wards with larger amounts of *pig farming* were at a higher risk of *death* than wards with no *pig farming*.

***Poultry*** represented the total number of poultries in each ward, from the farm census. This study surmised that wards with larger amounts of *poultry farming* were at a higher risk of *death* than wards with no *poultry farming*.

The livestock variables (number of *cattle, pigs,* and *poultry* within each ward) were rescaled from large continuous variables into four-level categorical variables (scored 0–3). Wards with 0–3 head of each livestock variable were recoded as 0 to represent *none of that animal* within that ward. The remaining amount of each livestock variable was divided by 3 to create equally banded categories across wards, representing small (1), medium (2), and large (3) quantities of each animal per ward.

### Dataset compilation

The Statistical Package for Social Science (SPSS, Version 25) software was used to construct the three mortality-based datasets (IBM Corp, 2017). The ward-level variables were individually extracted in count form *via* an Excel spreadsheet, imported into SPSS-25 to collate the data, and then imported into MPlus 8.1 (Muthén and Muthén, 1998-2019) for further analysis (see supplementary material: NINIS data extraction procedure). The three datasets were set up in wide format. Geographical locations of wards were used as the linking identifier *via* a deterministic one-to-one matching algorithm (Zhu et al., 2015). The farm census variables formed the spine of the 2001 and 2011 datasets. All other variables from the population census were downloaded in an identical format to ensure harmonious merging and configuration. Data cleaning and missing value analyses indicated that there were no missing data present within the variables (Dziadkowiec et al., 2016).

### Strengths

The NINIS digital ecosystem stores agricultural and administrative records (collected routinely for different purposes) independently and in isolation, which minimises the usefulness of the data. The current study provides evidence that the NINIS information is findable, accessible, interoperable, reusable, and expandable (Table 1). The innovative design of these datasets encourages future researchers to utilise publicly available macro and/or micro data to unlock secondary value from the data.

This paper presents information on NINIS data in a way that is accessible to readers who may not share the author’s particular specialism. In addition, this research will encourage future investigators to conduct interdisciplinary research as no other study has applied this approach, combining NINIS agricultural (agro-ecological science) and administrative data (health science) to facilitate new research questions within NI wards (Morrison et al., 1993). Conclusions drawn from these multiple data sources will be stronger with a reduced risk of error, in contrast to those drawn from only a single source.

This study used the two most up-to-date population censuses (2001 and 2011) and then collated the farm census data from the same years. The two distinct periods (2001 and 2011) covered identical areas but are not necessarily the same people, while the 2011 database provides the most up-to-date information currently available. This paper provides the methodology on how to analyse the next census once it becomes available online. However, the current census for 2021 has been delayed due to COVID-19 and has been rescheduled for enumeration in April 2022. The authors of this study intend to merge the next census information (once available on the NINIS website) to the current datasets.

The adjustment of the SGM to SO in 2011 does not affect the current study’s reproducibility. Therefore, an extension of this study may utilise SGM (2001 and 2010) and SO (2011 and 2021) variables to compare the differences in farming outputs over 10 years as this would allow one to analyse the changes in the agricultural economic size between wards. NI policymakers within the Department of Agriculture, Environment, and Rural Affairs (DAERA), National Health Service (NHS), and Health and Safety Executive Northern Ireland (HSENI) could collaboratively use these datasets to identify agriculturally based wards (with higher deaths in 2011 than in 2001) and tailor farmers’ healthcare needs accordingly.

### Limitations

As a result of NISRA’s aggregation procedures, a small amount of data loss occurred during the summarisation of information. Moreover, the three datasets within this paper do not possess the ability to analyse the associations of individual-level variables, as the information used for compilation was aggregated (or clustered) at the ward level. Lokar et al. (2019) stated that this means researchers must be cautious to avoid making an *ecological fallacy* when interpreting results, bearing in mind that individual- and ward-level predictor associations with deaths are different (Robinson 1950). Jacob (2016) reported that some researchers remain hesitant to analyse the aggregated data as there is an underlying misconception that clustered data have restricted use; however, NINIS aggregated data results will provide the benefits of efficiency and power. Moreover, despite these variables being aggregated at the ward level, there were robust and vast amounts of literature supporting their use within this study of mortality (Haan et al., 1987; Yen & Kaplan, 1999; Chan et al., 2014).

## Conclusion

In order to fill the gaps in the current research, this data report collated the NINIS information at ward level from the population and farm censuses (2001 and 2011) to compile three mortality-based datasets (NISRA 2019). The 2001 and 2011 mortality datasets were compiled for future cross-sectional analyses and subsequently pooled into a third dataset for longitudinal analyses. This study expanded on the design, methodology, and analytic limitations of a neighbourhood social environment study conducted by Yen and Kaplan (1999). This NI study was conducted in response to: i) corresponding reductions in the number of deaths of farmers between census years and ii) no nationally representative datasets available with the ability to analyse economic, environmental, and social influences on mortality patterns between agricultural and non-agricultural wards.

The purpose of this paper is to aid researchers who would like to use these datasets for future analyses by making the methodology that underpins it transparent and easy to replicate. For example, future researchers can use these data to investigate whether agriculturally saturated wards have a higher mortality risk than non-agriculturally based wards of NI. For didactic purposes, this data report provides the descriptive, structural, and administrative metadata (Davenhall 2011) of the datasets so that external investigators who want to replicate, reuse, adapt, or expand it can do so competently by utilising this approach.

This data report is of interest to the One Health research community as it standardizes the environment−human−animal data to pave the way towards a new One Health research paradigm. In addition, these three datasets use novel data linkage methods, and their integrative analyses will enhance the understanding of the interconnectedness of the environment and multiple species within NI. Furthermore, the current amalgamation of data within this paper allows for an early release of summary results, alongside an extensive array of academic research uses.

## Data Availability

The original contributions presented in the study are included in the article/Supplementary Material; further inquiries can be directed to the corresponding author.
