# Supplementary material for: Data report on three datasets: Mortality patterns between agricultural and non-agricultural ward areas
Source: Front Genet. 2023 Jan 4;13:953167. doi: 10.3389/fgene.2022.953167 (PMC9851396; doi:10.3389/fgene.2022.953167)
Supplement: Supplementary file 1 [file Table1.DOCX]

**NINIS Data Extraction Protocol**

How to extract NINIS data via online databank via table 1 codes (NISRA 2019).

**Retrieving data from NINIS databank converting into Excel documents**

1. Enter 2001 or 2011 variable name or code into NINIS search bar from (table 1)
2. View information by: *(geography)* ward; *(view by)* geography code and name display; *(year)* 2001 or 2011; *(variables)* select all; and *(export as)* .*csv*
3. Open new Excel document downloaded above

- Change variable names to have no spaces, remove time point year at end of names, ensure all data are in numeric form
- Delete redundant lines: first horizontal line with total *“Northern Ireland”* figures, horizontal lines with ward descriptor information, and any empty vertical columns
- Get rid of all commas within dataset by highlighting all numbers within file then:

Home > across to *‘number’* within drop down menus (e.g., general, number, currency, etc.) click *number* (making all commas within the numbers disappear)

- Decrease six decimal places in all *.csv* file to only three places

**Importing data from Excel files into SPSS Version 25**

1. File > import data > as *.csv* data > select file created above by name > *(type)* *.csv* file, *(encoding)* local encoding > open

- Read .*csv* file > (tick) first line contains variable names > *(delimiter between values)* comma, *(decimal symbol)* period, *(test qualifier)* double quote
- *.csv* format of excel document has been successfully imported into SPSS software with first line (or names) in excel now used as variable names in SPSS data file and all variables directly readable by SPSS (all scaled as continuous, change scale types accordingly)
- All missing values make -99 numeric number (data > transform > recode same variables > select all variables > SYSMIS -9 > ok) as it makes file fully complete in order to be read correctly in software (preventing future warnings because of missing spaces)

1. Perform deterministic linkages (full outer one-to-one join) ^11^ using ward identifiers, adding each variable [1] together to compile the 2001 and 2011 datasets
2. Transform all variables accordingly for specific analyses conducting
3. Only keep variables in SPSS final dataset using in the Mplus models (remove those not using for succinct Mplus syntax writing)

**Exporting data from SPSS and importing into Mplus Diagrammer 8**

1. File > save SPSS data as >

- *(tab delimited)* .*dat* file, needed for Mplus to read or import into its *.inp* for syntax writing and analyses
- *(tick)* *“keeping 26 of 26 variables”* file name “new name here”
- *(untick)* ‘write variable names in text file’
- *(untick)* ‘save value labels where defined instead of data values’
- *‘Encoding’* leave blank to use default of *‘local encoding’*

1. Save new *.dat* file to specified location on computer

**Using NINIS data in Mplus**

1. Open .*dat* file in Mplus8 Editor or Diagrammer to ensure full data file are numeric only with no variable names, check entire data file for any foreign symbols or spaces
2. Check this text version of the data looks the way it is expected to look
3. Make sure file has a) no headings; b) no text, only numbers; c) no unusual or foreign characters anywhere within it (e.g. on first/last line; d) there is not an extra line at the end of the data file (if so, delete this line and resave); e) ensure file structure has same layout as SPSS .sav file (wide or long)
4. *NINIS Files are ready to be analysed in Mplus*

**Optional Steps**

1. Increase or decrease Mplus syntax window sizes by bottom right corner (diagonal arrow) on opened input file window
2. To begin Mplus reading the NINIS data:

- Mplus syntax writing is more manageable if data (*.dat*) file kept in same folder as Mplus syntax or input *(.inp)* file
- If *.dat* or data file not in same folder as *.inp* input file, a path needs specified; for example on desktop > desktop > right click on file and press alt > left click *‘copy data pathname’* > paste this pathname into Mplus in the data statement > (change direction of all slashes) from a slash / to a backslash \ (button above enter) and put in what *.csv* file is called

*DATA: file = /Users/Desktop/pathname.dat ;*

1. To begin Mplus analyses:

- Enter *.dat* file name in Mplus syntax (*.inp*)
- *DATA: file is "MplusFILEname.dat"*
- Keep *‘variable names’* in Mplus syntax (*.inp*) below 8 characters and keep all lines of syntax below 90 characters
- Options > select font > *(size)* 14 and *(font)* courier > easier to read syntax and output

1. If using Mplus Diagrammer:

- Once model is run
- Alongside model outputs, it also provides a window with the model displayed graphically
- Can move around components of this diagram, for ease of visual interpretation
